# A Simple and Efficient Molecularly Imprinted Electrochemical Sensor for the Selective Determination of Tryptophan

**DOI:** 10.3390/biom9070294

**Published:** 2019-07-22

**Authors:** Yaling Tian, Peihong Deng, Yiyong Wu, Ziyu Ding, Guangli Li, Jun Liu, Quanguo He

**Affiliations:** 1 School of Life Sciences and Chemistry, Hunan University of Technology, Zhuzhou 412007, China; 2 Hunan Provincial Key Laboratory of Functional Metal-Organic Compounds; Key Laboratory of Functional Organometallic Materials of Hunan Provincial Universities; Department of Chemistry and Material Science, Hengyang Normal University, Hengyang, Hunan 421008, China

**Keywords:** molecularly imprinted chitosan film, electrochemical sensor, tryptophan, selective determination

## Abstract

In this paper, a tryptophan (Trp) molecularly imprinted chitosan film was prepared on the surface of an acetylene black paste electrode using chitosan as the functional polymer, Trp as the template molecule and sulfuric acid as the crosslinking agent. The surface morphologies of non-imprinted and imprinted electrodes were characterized by scanning electron microscopy (SEM). The formation of hydrogen bonds between the functional polymer and the template molecule was confirmed by infrared spectroscopy. Some factors affecting the performance of the imprinted electrode such as the concentration of chitosan, the mass ratio of chitosan to Trp, the dropping amount of the chitosan-Trp mixture, the solution pH, and the accumulation potential and time were discussed. The experimental results show that the imprinted electrode exhibit good affinity and selectivity for Trp. The dynamic linear ranges of 0.01–4 μM, 4–20 μM and 20–100 μM were obtained by second derivative linear sweep voltammetry, and the detection limit was calculated to be 8.0 nM. The use of the imprinted electrode provides an effective method for eliminating the interference of potentially interfering substances. In addition, the sensor has high sensitivity, reproducibility and stability, and can be used for the determination of Trp in pharmaceutical preparations and human serum samples.

## 1. Introduction

Tryptophan (Trp) is an important amino acid for the human body. Low concentrations of Trp are essential for many physiological functions. It is a precursor of serotonin and melatonin that significantly improves the mood, sleep and mental health. It is also an important component of protein, capable of establishing and maintaining a positive nitrogen balance [1]. Because Trp is seldom found in vegetable products, this compound is sometimes added to diets, food, and pharmaceutical formulations. However, if Trp is excessively consumed or cannot be properly metabolized, high concentrations of Trp would have some harmful effects on the human body, such as producing a toxic waste in the brain, leading to hallucinations and delusions [2]. In addition, it may be a cause of schizophrenia for people. Because of its small thresholds between essential and toxic levels in living organisms, it is of great clinical significance to determine Trp simply, accurately and quickly. Besides, Trp often coexists with ascorbic acid (AA), dopamine (DA), uric acid (UA), nitrite (NO_2_^−^) and other amino acids in biological matrixes. Therefore, the selective determination of Trp is also very important.

At present, there are many analytical methods for Trp determination, such as liquid chromatography [3], gas chromatography [4] and thin layer chromatography [5]. Chromatographic techniques are highly selective and accurate, however, they often require pretreatment steps, expensive and complicated instruments, skilled operators as well as consume more time. Some non-chromatographic methods such as spectrophotometry [6], fluorescence [7] and chemiluminescence [8] are also used to determine Trp. However, they suffer from low selectivity. Derivative spectroscopy [9] and multivariate calibration techniques [10] are sometimes used to solve the problem of poor selectivity. Electroanalytical techniques have great appeal in monitoring biologically important molecules due to their simplicity, low cost, portability and sensitivity [11,12,13,14,15]. Because Trp can be oxidized on the electrode, recent studies have focused on the determination of Trp by electrochemical methods. However, the direct oxidation of Trp on bare electrodes results in slow electron transfer and high overpotentials [16]. Some chemically modified electrodes have been reported for the determination of Trp [16,17,18,19,20,21,22,23,24,25,26,27], and comparative analytical figures of merit for different electrodes are summarized in Table 1. Although these sensors can offer high sensitivity and a low detection limit, they suffer from the main drawback, which is the moderate selectivity. According to these studies, the oxidation peak potentials for some electroactive biomolecules, such as ascorbic acid (AA), uric acid (UA), dopamine (DA) and tyrosine (Tyr), are very close to that of Trp, and the voltammetric responses of Trp and these substances exhibit severe overlapping, which makes their practical applications limited. Since Trp often coexists with these biomolecules in food processing, pharmaceutical formulations and biological fluids, to determine Trp selectively based on its electroactivity is of great challenge.

In recent years, molecularly imprinted polymers (MIPs) have been widely used in various fields such as artificial antibodies, chemical sensors and solid phase extraction, due to their excellent selectivity, good chemical and physical stability, as well as low cost [28,29,30]. There are also increasing reports of using MIPs to modify the electrode surface to enhance selectivity [31,32,33,34]. In general, molecular imprinting is the process of polymerizing selected functional monomers around the template molecules in the presence of a cross-linking agent. After polymerization, the template molecules are extracted to obtain a polymer matrix that is complementary in shape and functionality to the template. Thus, the polymer has the ability to selectively attach to the analyte. Chitosan is a natural polymer (a polysaccharide) obtained by the partial deacetylation of chitin. Because of its excellent film forming ability, biocompatibility, biodegradability, lack of toxicity and low cost, chitosan has been widely used in membrane separation, drug delivery and environmental protection [35,36,37]. In addition, chitosan can chelate and adsorb with many substances based on a large number of hydroxyl groups and amino groups. In recent years, chitosan has also been reported for the construction of imprinted electrochemical sensors and shows good selectivity [38,39,40,41]. For example, Guo and his colleagues fabricated a novel molecularly imprinted electrochemical sensor modified with carbon dots, chitosan, and gold nanoparticles for the detection of patulin. The linear response range of the MIP sensor was from 1.0 pM to 1.0 nM and the limit of detection (LOD) was 0.757 pM [38]. Wu et al. developed an electrochemical sensor based on ion-imprinted chitosan-graphene nanocomposites for the sensitive and selective determination of Cr (VI). The linear range of the MIP sensor was from 1.0 nM to 10 μM, with a low detection limit of 0.64 nM [39]. Liu et al. constructed a MIPs electrochemical sensor based on graphene-chitosan composite and used in dopamine measurements. The linear range was from 1.0 nM to 80 nM and 0.1 μM to 100 μM. The sensor exhibited high selectivity for the determination of dopamine in the presence of some structural analogues and coexisting interferences [40]. Xia and his colleagues prepared a novel protein molecularly imprinted electrochemical sensor based on a chitosan/ionic liquid–graphene modified glassy carbon electrode via electrochemical polymerization, which could be used for the sensitive and selective detection of bovine serum albumin [41]. However, as far as we know, the design and fabrication of imprinted electrochemical sensors for Trp detection using chitosan have not been reported in any literature.

Many applications require not only better selectivity, but also higher sensitivity. Acetylene black (AB) has become an effective sensing platform for the development of electrochemical sensors and biosensors in recent years because of its unique advantages such as large specific surface area, good chemical stability, high mechanical strength and excellent conductivity [42,43]. In this paper, an electrochemical sensor with high sensitivity and selectivity for the voltammetric determination of Trp was developed by combining acetylene black with the molecular imprinting technique. Using natural chitosan as functional polymer and Trp as template molecule, Trp molecularly imprinted chitosan film was prepared on the surface of an acetylene black paste electrode (ABPE). Various factors affecting the electrochemical performance of the imprinted electrode were investigated in detail. The new sensor has the characteristics of good selectivity, simple preparation and easy operation. Finally, this MIP/ABPE was successfully applied to the quantitative analysis of Trp in pharmaceutical preparations and human serum samples.

## 2. Experimental

### 2.1. Chemicals and Solutions

Trp and other amino acids, uric acid, ascorbic acid, dopamine, sodium nitrite, oxalic acid, glucose, lactic acid, and tartaric acid were purchased from Aladdin Chemical Reagent Co., Ltd., Shanghai, China. Acetylene black (AB, purity > 99.99%) was purchased from STREM Chemicals, USA. Chitosan (95% deacetylation) was purchased from Shanghai Biochemical Co. Ltd., China. The human blood serum samples were obtained from a local hospital. The standard stock solution (1.0 × 10^−3^ M) of Trp was prepared by dissolving Trp into 0.1 M HCl and diluting to 100 mL with water, which was stored at 4 °C and was stable for two weeks. The working solution was freshly prepared before use by diluting the stock solution. All other reagents were of analytical grade and used directly. The water used was doubly distilled.

### 2.2. Apparatus

The morphologies of the non-imprinted and imprinted films were observed by a scanning electron microscope (EVO10, ZEISS, Jena, Germany). Fourier transform infrared spectroscopic measurements were performed on an IRPrestige-21 Fourier transform infrared spectrometer (Shimadzu Corp., Tokyo, Japan). Cyclic voltammetry (CV) was carried out on a CHI 660D electrochemical workstation (Chenhua Instrument Co. Ltd., Shanghai, China) controlled by a microcomputer with CHI660 software. A model JP-303E polarographic analyzer (Chengdu Instrument Factory, Chengdu, China) was used to give the second-order derivative linear sweep voltammograms for electroanalytical measurements. A three-electrode system was used thoughout the electrochemical measurements, where the MIP/ABPE was used as the working electrode, a platinum wire as the counter electrode and a saturated calomel electrode (SCE) as the reference electrode. All potentials reported were versus the SCE. pH measurements were performed with a pH-3c Model pH meter (Shanghai Leichi Instrument Factory, Shanghai, China) using a combined glass electrode.

### 2.3. Preparation of MIP/ABPE

**Scheme 1:**
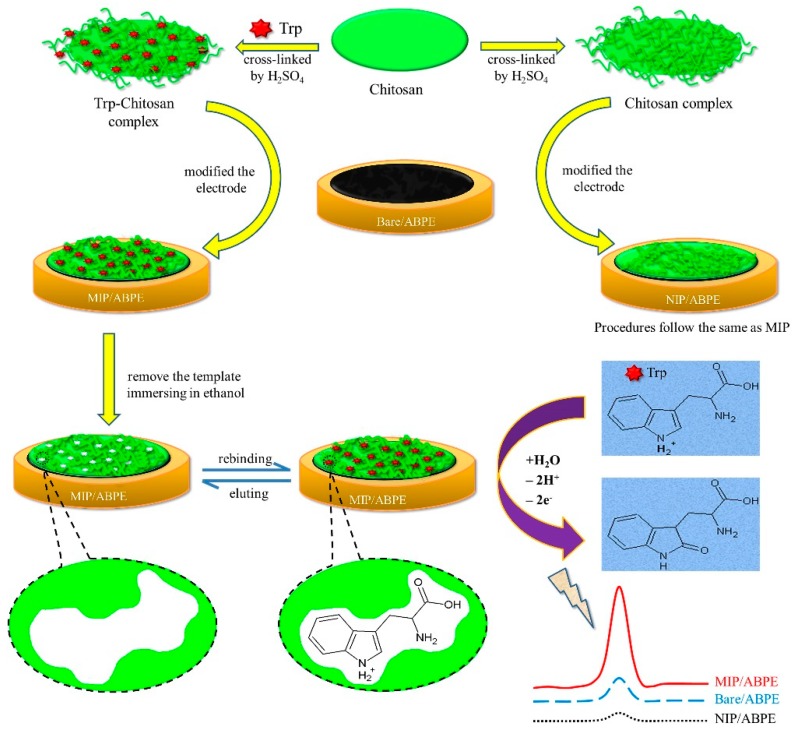
The procedure for fabrication of the molecularly imprinted polymers (MIP)/acetylene black paste electrode (ABPE).

A total of 1.20 g of acetylene black powder and 0.30 g of solid paraffin were ground uniformly in a mortar. Subsequently, the mixture was heated to 75–80 °C to melt the solid paraffin. The homogenous paste was tightly pressed into an electrode tube (1.0 mm in diameter and 3.0 mm in depth) while it was hot. The surface of the electrode was polished on a weighing paper to remove the excess of solidified material. For the preparation of the MIP/ABPE, 50.0 mg of chitosan was dissolved in 10.0 mL 1.0% (*v*/*v*) acetic acid aqueous solution. At the same time, a certain amount of template molecule Trp was added. The mixed solution was stirred at room temperature for 4 h. Subsequently, 5 μL of the obtained solution was drop-coated onto the ABPE surface and the solvent was left to evaporate under ambient conditions. Then the chitosan film was cross-linked by immersing the modified electrode into a 0.5 M sulfuric acid solution at room temperature for 24 h [44]. After that, the modified electrode was subjected to a washing procedure by repetitive immersion in ethanol to remove the Trp template entrapped in the polymeric matrix, and then air-dried for 24 h. The obtained imprinted electrode was tagged as MIP/ABPE. The entire process of electrode preparation was shown in Scheme 1. As a control, the non-imprinted electrode (NIP/ABPE) was prepared with the same procedure just without adding the template molecules. In order to show their unique properties, AB, MIP/CPE (CPE refers to carbon paste electrode throughout the following text) and NIP/CPE, were also prepared in a similar manner, only graphite powder was needed to replace acetylene black powder.

### 2.4. Electrochemical Measurements

All electrochemical experiments including cyclic voltammetry (CV) and second-order derivative linear sweep voltammetry were carried out with a standard three-electrode system, using bare or modified electrodes, a platinum wire electrode, and a saturated calomel electrode (SCE) as the working electrode, counter electrode and reference electrode, respectively. The CV was used for the electrode characterization in a solution consisting of 1.0 mM K_3_[Fe(CN)_6_] and 0.5 M KCl. The sensing performance of Trp on MIP/ABPE and the optimization of measuring conditions, as well as the selectivity, reproducibility and stability experiments, were carried out by second-order derivative linear sweep voltammetry. A 10.0 mL volume of the solution, containing an appropriate concentration of Trp and 0.1 M phosphate buffer (pH 7.0) was transferred into a voltammetric cell. The stirrer was switched on. The accumulation potential of −0.1 V was applied to the MIP/ABPE for 180 s. Following the accumulation period, the stirrer was stopped, and after a rest period of 5 s, the second-order derivative voltammogram was recorded by applying a positive-going potential scan from 0.2 to 1.2 V at 0.1 V s^−1^, and the second derivative peak of Trp was obtained at about 0.756 V. After each measurement, the imprinted electrode was immersed in a 0.1 M phosphate buffer (pH 7.0) and treated with repetitive potential scanning from 0.2 to 1.2 V at a scan rate of 0.1 V s^−1^ to remove the template molecules until the baseline became stable. To demonstrate the MIP/ABPE repeatability, the results were averaged for three measurements at the same electrode. All the measurements were performed at room temperature.

## 3. Results and Discussion

### 3.1. Template Removal

**Figure 1:**
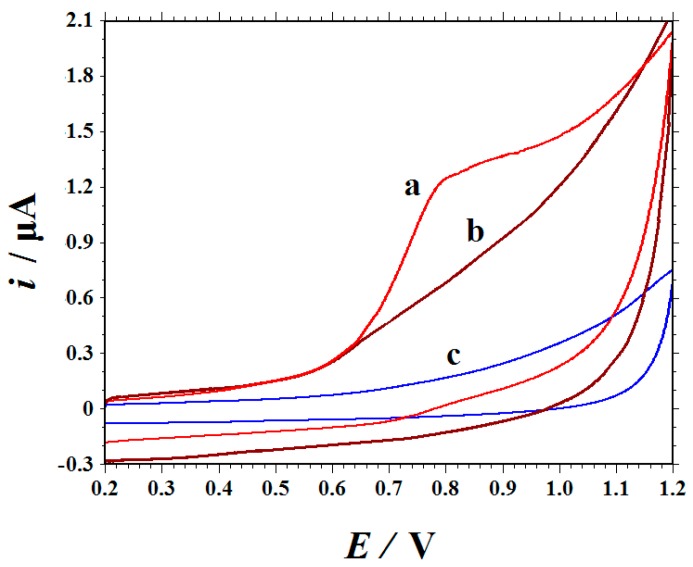
The cyclic voltammograms in the 0.1 M phosphate buffer (pH 7.0) recorded on the molecularly imprinted polymers (MIP)/acetylene black paste electrode (ABPE) before extraction (curve a) and after extraction (curve b), non-imprinted electrode (NIP)/ABPE (curve c). Scan rate: 0.1 V s^−1^.

Figure 1 shows the CVs of the MIP/ABPE and NIP/ABPE in a 0.1 M phosphate buffer (pH 7.0) with the potential ranging from 0.2 to 1.2 V. Before extracting the template molecules, a broad oxidation peak at about 0.80 V could be seen (curve a). Because the electrochemical measurement was carried out in a Trp-free solution, this means that the oxidation peak was caused entirely by Trp embedded in the chitosan film. When MIP/ABPE was immersed in ethanol and the Trp template molecules were removed from the chitosan matrix, almost no electrochemical response is observed, as shown in curve b. The disappearance of the CV signal indicates that Trp is effectively removed. No peaks are observed at the NIP/ABPE (curve c).

### 3.2. FT–IR Spectra

**Figure 2:**
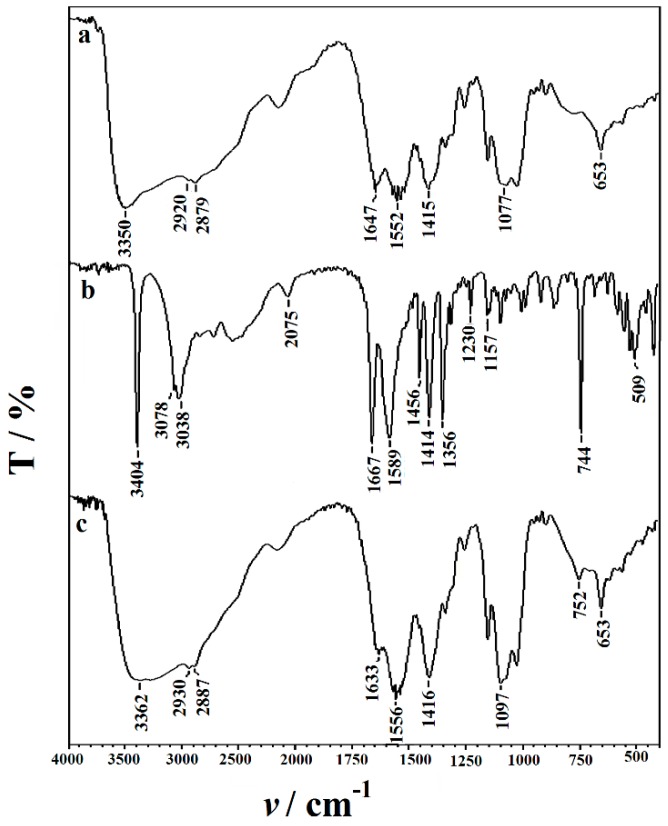
The Fourier Transform Infrared (FT–IR) spectra of (**a**) chitosan, (**b**) Trp and (**c**) the Trp-chitosan composite.

Figure 2 displays the Fourier Transform Infrared FT–IR spectra of chitosan, Trp and the chitosan-Trp composite. The spectrum of chitosan (Figure 2a) showthe absorption peaks at about 3350 cm^−1^ for the –NH_2_ and –OH groups, at about 2920 and 2879 cm^−1^ for the aliphatic C–H stretching vibration, 1647 cm^−1^ for the absorption peak of the rest NH_2_CO group, and 1077 cm^−1^ for the C–O group. Figure 2b demonstrates the FT-IR of Trp. A strong and sharp peak is observed at 3404 cm^–1^, which corresponds to the N–H stretching in the indole group of Trp, and two poor resolved bands between 3078–3038 cm^−1^ correspond to the asymmetric and symmetric stretching modes of the amino group. IR bands observed at 1667 and 1589 cm^−1^ correspond to the COO^-^ and NH_3_^+^ asymmetric stretching vibrations, respectively. The peaks at 1456, 1414 and 1356 cm^−1^ are assigned to the NH_3_^+^ symmetric stretching vibration, the COO^−^ symmetric stretching mode and the C–H deformation mode, respectively [45]. In Figure 2c, the broaden absorption peak at about 3500–3000 cm^−1^ corresponds to the hydrogen bond strength. In comparison with that of Figure 2a, the C=O absorption peaks moved from 1647 to 1633 cm^−1^. These changes are due to the formation of hydrogen bonds between the functional polymer and the template molecule.

### 3.3. Electrode Characterizations by SEM and CV

**Figure 3:**
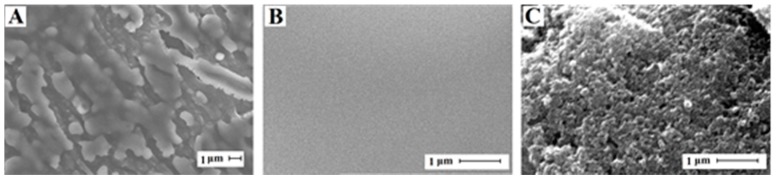
The scanning electron microscopy (SEM_ images of (**A**) ABPE, (**B**) NIP/ABPE and (**C**) MIP/ABPE.

The surface morphologies of ABPE, NIP/ABPE and MIP/ABPE were studied by scanning electron microscopy (SEM). As shown in Figure 3A, the surface of ABPE is rough and uneven, and AB particles show an irregular and large flake structure. The surface of NIP/ABPE is smooth and flat (Figure 3B), which is attributed to the formation of a compact chitosan film on the electrode surface. As illustrated in Figure 3C, the top view of MIP/ABPE changes significantly compared to NIP/ABPE. After removing the template molecules, a three-dimensional network porous structure is observed on the MIP/ABPE, indicating that the specific cavities are formed in the chitosan matrix.

**Figure 4:**
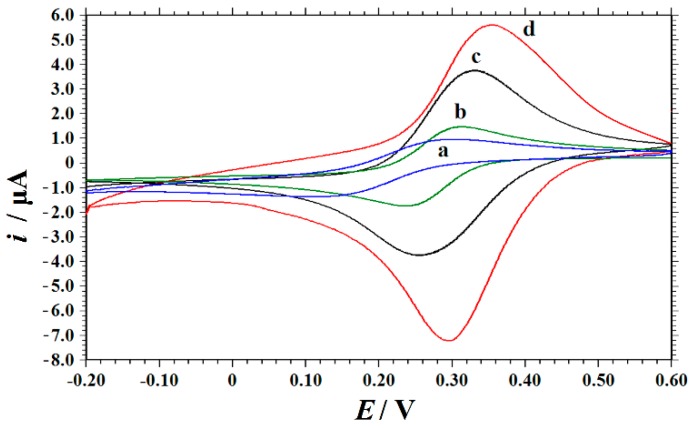
The cyclic voltammograms obtained at different electrodes in the 1.0 mM K_3_[Fe(CN)_6_] solution containing 0.5 M KCl. (**a**) CPE, (**b**) ABPE, (**c**) MIP/ABPE before extraction, (**d**) MIP/ABPE after extraction. Scan rate: 0.1 V s^−1.^

The cyclic voltammetric behaviours of 1.0 mM K_3_[Fe(CN)_6_] containing 0.5 M KCl at different electrodes were studied at a scan rate of 0.1 V s^−1^. Figure 4 shows the corresponding cyclic voltammograms obtained at CPE (curve a), ABPE (curve b), MIP/ABPE before and after extraction of the template molecules (curve c and cruve d), respectively. K_3_[Fe(CN)_6_] shows a pair of quasi-reversible CV peaks on bare CPE. The cathodic peak potential (*E*_pc_) is 111 mV, the anodic peak potential (*E*_pa_) is 302 mV, and the peak separation (Δ*E*_p_) is 191 mV. Compared with bare CPE, the peak current of K_3_[Fe(CN)_6_] on ABPE increases significantly and the peak separation decreases to 75 mV, indicating that AB can improve the electron transfer rate in the redox process of [Fe(CN)_6_]^3−/4−^. This is because AB has good conductivity and a large specific surface area, which provides a suitable bed for MIP immobilization. Before Trp extraction, the CVs of MIP/ABPE show two distinct redox peaks, the current is higher than that of ABPE, and the peak separation is 99 mV. This may be due to the affinity of positively charged chitosan to negative charge [Fe(CN)_6_]^3−/4−^ [46]. As compared with MIP/ABPE before Trp extraction, the current of MIP/ABPE after Trp extraction increases further. This can be explained because when Trp is extracted from the chitosan film, the three-dimensional imprinted cavities matching with Trp is formed not only in the spatial structure, but also in the functional groups. With these cavities, [Fe(CN)_6_]^3−/4−^ can easily reach the surface of ABPE through the chitosan film due to its small size. Therefore, the increase in current observed at MIP/ABPE is attributed to the imprinting characteristics.

### 3.4. The Imprinting Effect and the Electrode Process Mechanism

**Figure 5:**
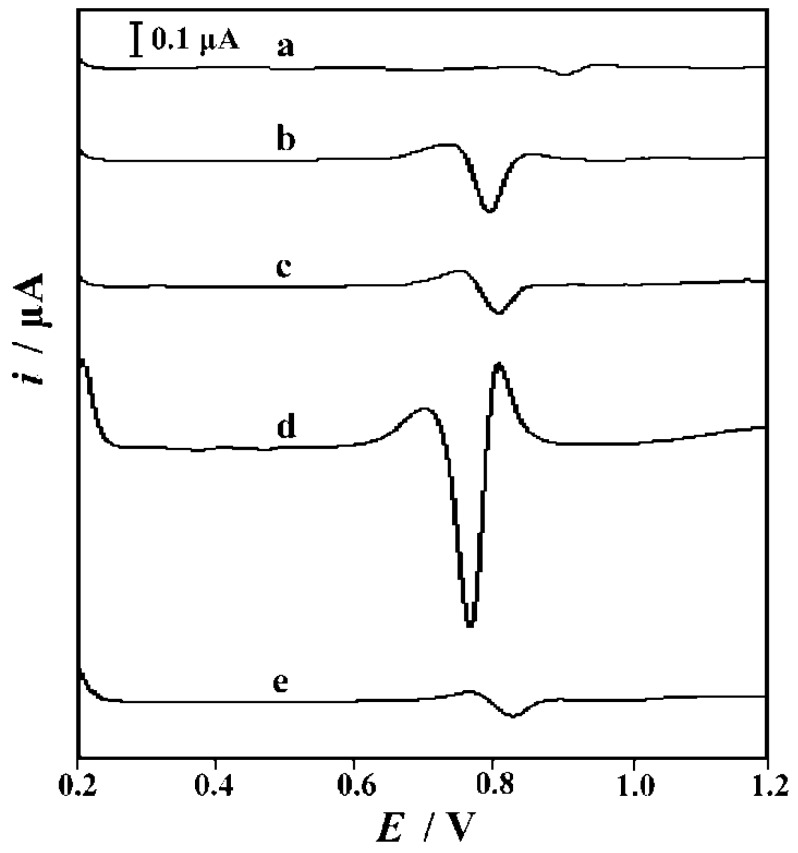
The second-order derivative linear sweep voltammograms of 0.1 mM Trp recorded at different electrodes: (**a**) CPE, (**b**) ABPE, (**c**) MIP/CPE, (**d**) MIP/ABPE and (**e**) NIP/ABPE in 0.1 M phosphate buffer (pH 7.0). Accumulation potential: −0.1 V, Accmulation time: 60 s, scan rate: 0.1 V s^−1^.

The extraction of Trp results in the formation of sites in the chitosan matrix that can selectively recombine the template molecules. The affinity and selectivity of the imprinted electrode were evaluated by second derivative linear sweep voltammetry. In comparison with differential pulse voltammetry (DPV) and square wave voltammetry (SWV), the second derivative linear sweep voltammetry has a higher current, better signal to background characteristics and better resolution of overlapping [42,43]. Figure 5 shows the second derivative linear sweep voltammetry of Trp oxidation at CPE (curve a), ABPE (curve b), MIP/CPE (curve c), MIP/ABPE (curve d) and NIP/ABPE (curve e) after accumulation at −0.1 V for 60 s in a 0.1 M phosphate buffer (pH 7.0) containing 0.1 mM Trp. Obviously, CPE has the lowest peak current (914 mV, 0.02781 µA). When ABPE is used, the peak current of Trp increases (796 mV, 0.1953 µA) due to the specific surface area and special electrical performance of AB. The current responses of MIP/CPE (804 mV, 0.1256 µA) and MIP/ABPE (756 mV, 0.8226 µA) are significantly higher than those of CPE and ABPE, which could be attributed to the existence of MIP. Because of the inherent three-dimensional cavities formed in the MIP, more Trp molecules were selectively adsorbed on the specific recognition sites by rebinding groups, and the current response increases greatly. At the same time, the current response of MIP/ABPE is about 6 times that of MIP/CPE, which further confirm the current amplification effect of AB. Conversely, the significant decrease of peak current on NIP/ABPE (0.06313 µA) can be attributed to the absence of specific binding sites and cavities in the NIP. According to the maximum peak current obtained on MIP/ABPE, MIP/ABPE can be used as a novel sensor for Trp detection with high sensitivity and selectivity.

**Figure 6:**
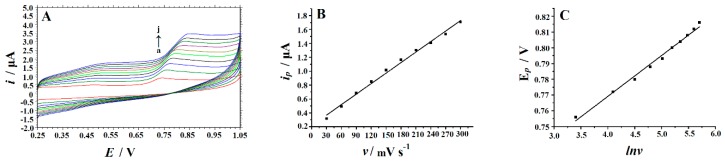
(**A**) The cyclic voltammograms of 0.1 mM Trp in 0.1 M phosphate buffer (pH 7.0) obtained on the MIP/ABPE at different scan rates (a–j: 30, 60, 90, 120, 150, 180, 210, 240, 270, 300 mV·s^−1^); (**B**) The plot of the peak current versus the scan rate; (**C**) The plot of the peak potential versus the Napierian logarithm of the scan rate. Accmulation potential: −0.1 V; Accmulation time: 60 s; scan rate: 0.1 V s^−^^1^.

By analyzing the relationship between the oxidation peak potential or current of Trp and the scanning rate, useful information concerning the electrochemical mechanism of Trp can be obtained. The effect of the scan rate ranging from 0.03 to 0.3 V s^−1^ on the electrochemical performance of Trp was evaluated by cyclic voltammetry (CV) on MIP/ABPE and the results were shown in Figure 6. It was found that Trp produced only one oxidation peak on MIP/ABPE, which indicated that the electrode reaction of Trp was completely irreversible. With the increase of the scan rate, The peak current of Trp increased linearly with the increase of the scan rate (*i* = 0.0051 *v* + 0.2099 (*i*: μA, *v*: mV s^−1^), R^2^ = 0.9955), indicating that Trp is an adsorption control process on MIP/ABPE. The relationship between the oxidation peak potential and scan rate was also investigated, and it can be described as *E*_p_ = 0.02594 ln*v* + 0.6365 (*E*_p_: V, *v*: mV s^−1^), *R*^2^ = 0.9949. According to Laviron’s theory [47], the slope was equal to RT/α*n*F. The calculated value of *αn* was 0.9893. For the completely irreversible electrode reaction process, *α* is assumed to be 0.5. On the basis of the above discussion, *n* was calculated to be 1.98, indicating that there are two electrons involved in the oxidation of Trp on MIP/ABPE.

### 3.5. Optimization of Analytical Conditions

All the following analysis conditions were optimized using the second-order derivative linear sweep voltammetry. The scanning potential range was 0.2~1.2 V, the scanning rate was 0.1 V s^−1^, the supporting electrolyte was 0.1 M phosphate buffer (pH 7.0) and the concentration of Trp was 0.1 mM.

#### 3.5.1. The Effect of the Concentrations of Chitosan

**Figure 7:**
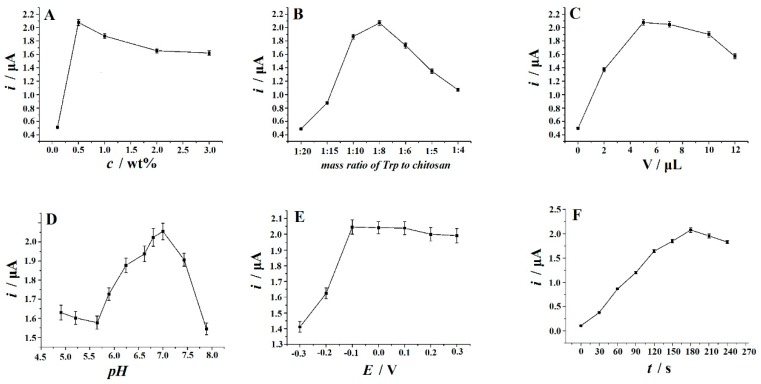
The effects of the concentration of chitosan (**A**), the mass ratio of Trp to chitosan (**B**), the dropping amount of Trp-chitosan suspension (**C**), the solution pH (**D**), the accumulation potential (**E**) and the accumulation time (**F**) on the oxidation peak current of 0.1 mM Trp at MIP/ABPE. When one parameter was changed, the other parameters were at their optimal values.

The effect of the concentration of chitosan on the response of Trp on MIP/ABPE was tested. Five different concentrations of chitosan (0.1 wt%, 0.5 wt%, 1.0 wt%, 2.0 wt%, 3.0 wt%) were examined. After accumulating at −0.1 V for 180 s in 0.1 M phosphate buffer (pH 7.0), it was found that the peak current of Trp increased with the increase of the concentration of chitosan up to 0.5 wt%. However, after the concentration exceeded 0.5 wt%, the peak current of Trp decreased. In the general condition, the viscosity of the chitosan solution increases with the increase of its concentration. A too high viscosity would induce a nonuniform thickness of the cast film; on the contrary, an unduly low viscosity would weaken the combination stability between the imprinting molecules and functional polymer, thereby debasing the selectivity of the imprinted electrode. Hence, 0.5 wt% of chitosan was selected as the optimum concentration (Figure 7A).

#### 3.5.2. The Effect of the Mass Ratio of Trp to Chitosan

The appropriate ratio of Trp molecule to chitosan determines the number of binding sites for selective recombination of Trp. In order to improve the sensing performance of MIP/ABPE, the ratio of Trp to chitosan was discussed. The effect of the mass ratio of Trp to chitosan was studied in the range of 1:4 to 1:20 (Figure 7B). After accumulating at −0.1 V for 180 s in a 0.1 M phosphate buffer (pH 7.0), the results show that when the ratio decreases, the peak current of Trp increases and reaches its maximum at 1:8. This is related to the change in the number of available binding sites. When the amount of chitosan is sparse, the number of binding sites available is small. However, a high concentration of chitosan might lead to a non-selective electrochemical response to the template. Here, the mass ratio of 1:8 was the best and was chosen for subsequent experiments.

#### 3.5.3. The Effect of Trp-Chitosan Dropping Amount

Different MIP/ABPEs were prepared by coating different volumes of Trp-chitosan on the surface of ABPE. Figure 7C showed the current response of 0.1 mM Trp on different MIP/ABPEs after accumulating at −0.1 V for 180 s in a 0.1 M phosphate buffer (pH 7.0). It was found that with the increase of the Trp-chitosan dropping amount, the peak current of Trp increased and the maximum current was obtained when the dropping amount reached 5 μL. However, the peak current of Trp decreased significantly when the dropping amount exceeded 5 μL. It is speculated that a very small dropping amount will reduce the number of effective imprinting sites on the surface of the electrode. A large dropping amount will lead to an increase in the film thickness, which will affect the conductivity of the electrode. For these reasons, the volume of Trp-chitosan suspension loaded on the ABPE surface was maintained at 5 μL.

#### 3.5.4. The Effect of the Solution pH

The effect of the solution pH on the peak current of 0.1 mM Trp at MIP/ABPE was studied in a 0.1 M phosphate buffer. After accumulating at −0.1 V for 180 s, the relationship between the oxidation current of Trp and the solution pH in the range of 4.91–7.89 was shown in Figure 7D. It was found that the oxidation current of Trp reached its maximum at pH 7.0. Therefore, pH 7.0 was chosen as the best pH value for the Trp measurement in the following experiments.

#### 3.5.5. Accumulation Potential and Accumulation Time

The effect of the accumulated potential on the peak current of 0.1 mM Trp was studied with a fixed accumulation time of 180 s in a 0.1 M phosphate buffer (pH 7.0). It can be seen that when the accumulation potential shifted from −0.3 V to −0.1 V, the peak current increases gradually and the peak current of Trp was kept almost constant at −0.1 V to 0.3 V (Figure 7E). Therefore, the best accumulation potential was chosen as −0.1 V. The effect of accumulation time on the oxidation peak current of Trp was also studied at a fixed accumulation potential of −0.1 V. It was found that the peak current of Trp increased with the increase of accumulation time. However, the oxidation peak current of Trp decreased slightly after 180 s (Figure 7F). Therefore, 180 s was chosen as the optimum accumulation time for Trp detection.

### 3.6. Analytical Performance of the MIP/ABPE

#### 3.6.1. Interference Study

**Figure 8:**
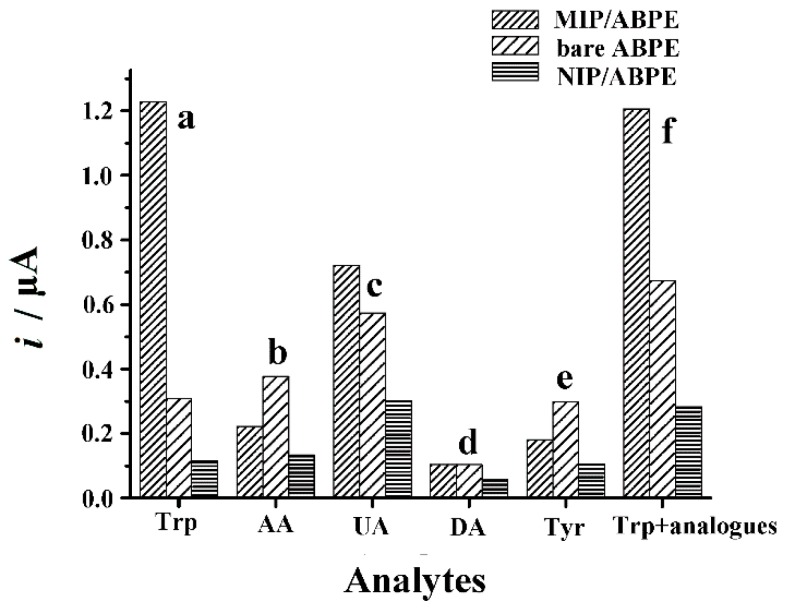
The selectivity of the MIP/ABPE: (a) 50 µM Trp; (b) 1 mM ascorbic acid (AA); (c) 1 mM uric acid (UA); (d) 1 mM dopamine (DA); (e) 1 mM tyrosine (Tyr); (f) 50 µM Trp mixed with 5 kinds of analogues (50 µM Trp + 1.0 mM AA + 1.0 mM UA + 1.0 mM DA + 1.0 mM Tyr). Supporting electrolyte: 0.1 M phosphate buffer (pH 7.0), incubation time: 120 s, scan rate: 0.1 V s^−1^.

The important performance of MIP sensors is the selective recognition of template molecules. In order to evaluate the selectivity of MIP/ABPE to Trp, interference experiments were carried out in the presence of ascorbic acid (AA), uric acid (UA), dopamine (DA) and tyrosine (Tyr), which usually coexist with Trp in biological fluids and pharmaceutical formulations. The results are shown in Figure 8. Obviously, the current response of Trp on the MIP/ABPE was higher than other substances. In addition, as shown in column e, the peak current of Trp did not change significantly after adding 20-fold concentrations of AA, DA, UA and Tyr into the Trp solution, but the current values of Trp changed greatly on bare ABPE and NIP/ABPE. Additionally, it was found that 100-fold concentrations of Na^+^, K^+^, Mg^2+^, Cu^2+^, Ca^2+^, Al^3+^, Pb^2+^, Cl^−^, NO_3_^−^, SO_4_^2−^, oxalic acid, citric acid, glucose, lactic acid, tartaric acid almost did not interfere with the Trp oxidation signal on MIP/ABPE (signal change was less than 5%). The effects of other amino acids such as glycine, alanine, valine, leucine, isoleucine, phenylalanine, histidine, aspartic acid, glutamic acid, lysine, arginine, serine, threonine, cysteine and proline on the determination of Trp were also studied. The results showed that any one of these amino acids (100 times content) did not interfere with the determination of Trp on MIP/ABPE. The results were summarized in Table S4 (Electronic Supplementary Material, ESM). The excellent selectivity might be attributed to the reason that the MIP/ABPE provided a thin imprinted polymer layer on the electrode surface. This layer had functional groups and selective cavities that specifically interacted with the template molecular Trp. During the imprinting process, the molecular space configuration was controlled by the large benzene ring in Trp molecules. After removing the imprinting molecules, the specific cavities were left in the chitosan network. the hydrogen bonds between chitosan and Trp improve the combination stability between the imprinting molecules and functional polymer, and the selectivity of MIP/ABPE.

#### 3.6.2. Linear Range and Detection Limit

**Figure 9:**
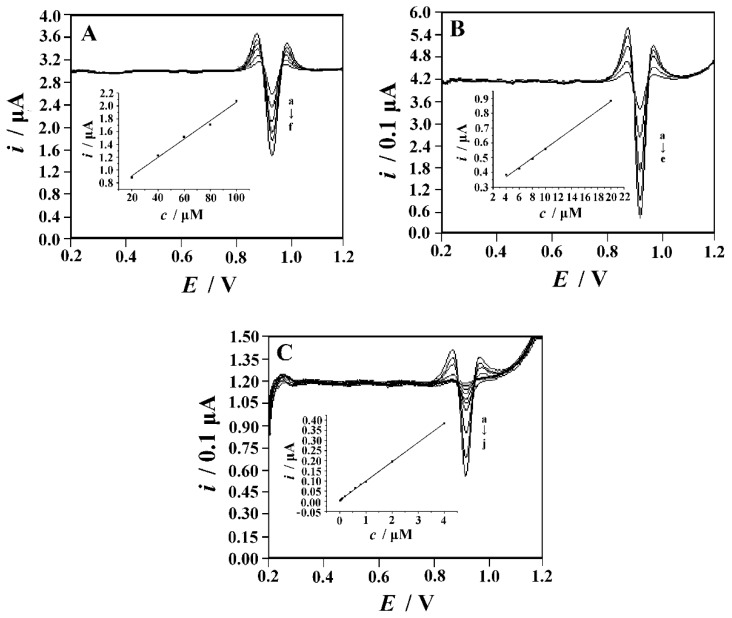
The second-order derivative linear scan voltammograms obtained at MIP/ABPE in a 0.1 M phosphate buffer (pH 7.0) and different concentrations of Trp: (**A**) from a to f : 10, 20, 40, 60, 80, 100 μM; (**B**) from a to e: 1.0, 2.0, 4.0, 6.0, 8.0 μM; (**C**) from a to j: 0.01, 0.02, 0.04, 0.06, 0.08, 0.1, 0.2, 0.4, 0.6, 0.8 μM, The inset of (**A**–**C**) shows the calibration curves of Trp in the range of 20–100 μM, 4–20 μM and 0.01–4 μM, respectively. Accumulation potential: −0.1 V, accumulation time: 180 s, scan rate: 0.1 V s^−1^.

The second derivative linear sweep voltammograms (Figure 9) show that the peak currents of Trp increase linearly with the increase of its concentration. Each point of the calibration plots corresponds to the average value obtained from three independent measurements. The calibration curve shows three linear regions (0.01–4.0 μM, 4.0–20 μM and 20–100 μM) in this relationship. The linear regression equations for these ranges were *i* (μA) = 0.0953*c* (μM) + 0.0044, *i* (μA) = 0.0319*c* (μM) + 0.243 and *i* (μA) = 0.0144*c* (μM) + 0.6212 with *R*^2^ = 0.9998, 0.9989 and 0.9960 respectively. The detection limits (LOD) were estimated by the formula of LOD = 3 s/m, s is the standard deviation of intercept and m is the slope of the regression line in the low concentration range [48]. The LOD of Trp were calculated to be 8.0 nM. The performance values of the developed sensor with other electrodes for Trp determination are compared in Table 1. From the linear range and detection limit, the performance of MIP/ABPE is superior to or comparable to other reported electrodes. In addition, the simple preparation process, low cost, high selectivity and environmentally friendly materials make the MIP/ABPE attractive in the Trp analysis.

#### 3.6.3. Reproducibility, Reusability and Long-Term Stability

Reproducibility and reusability are another two critical parameters for evaluating the performance of a sensor. The current response of 0.1 mM Trp on the same MIP/ABPE was measured repeatedly in a 0.1 M phosphate buffer (pH 7.0). After each measurement, the imprinted electrode was treated with repetitive potential scanning in a 0.1 M phosphate buffer (pH 7.0) to remove the template molecules. A relative standard deviation (RSD) of 1.70% (Table S2) was obtained (*n* = 7), which showed that the MIP/ABPE had good reusability. The reproducibility of the MIP/ABPE was determined by comparing the current responses of eight MIP/ABPEs, which were fabricated according to the same steps. An RSD of 4.18% (Table S3) was obtained, which showed that the reproducibility of the MIP/ABPE was acceptable. In addition, the stability of MIP/ABPE was also studied. In the first 10 days of storage, the response of the sensor to Trp did not deteriorate significantly. After 20 days of storage, the sensor maintained about 91% (Table S4) of its initial current response, indicating that the prepared MIP/ABPE had good long-term stability.

#### 3.6.4. Practical Application

To explore the practical application of MIP/ABPE, it was used to determine Trp in human serum samples and compound amino acid injections. A serum sample solution was prepared according to our previous report [11,49,50,51,52]. The compound amino acid injection was diluted 100 times with distilled water and used directly. A part of the sample solution was added to a 0.1 M phosphate buffer (pH 7.0). In order to prevent the matrix effect, the standard addition method was used to determine Trp content in samples. As shown in Table 2 and Table 3, the recoveries of human serum samples and amino acid injection samples are 97.3–102.4% and 98.3–104.2%, respectively. The results show that the electrode has high accuracy and selectivity for the determination of Trp in drugs and biological samples.

## 4. Conclusions

In the present paper, a molecularly imprinted chitosan film was prepared on the surface of an acetylene black paste electrode by the drop-coating method. After removing the template molecule, the molecularly imprinted membrane provides a highly tryptophan-affinity interface. Under the optimum conditions, a wider linear range and a lower detection limit are obtained. In addition, the imprint electrode has other advantages including simple fabrication, low cost, good repeatability and fast response; all of them make it hold great promise in the electro-analysis of Trp in real samples.

## Figures and Tables

**Table 1:** The comparison of the efficiency of molecularly imprinted polymers (MIP)/acetylene black paste electrode (ABPE) with other modified electrodes in the electrochemical determination of Trp.

Electrode	Technique	Supporting Electrolyte	Linear Range/μM	Detection Limit/μM	Effect of Tyr	References
^a^ BuCh/GCE	^m^ DPV	phosphate buffer (pH 7.0)	2–60	0.6	seriously interfered	[16]
^b^ Au-NPs/GCE	DPV	phosphate buffer (pH 2.5)	0.09–50	0.08	seriously interfered	[17]
^c^ nanoAu-MWCNTs/ GCE	DPV	phosphate buffer (pH 7.4)	5–100	3	seriously interfered	[18]
^d^ ETPGE	DPV	phosphate buffer (pH 3.0)	0.5–50	0.05	10-fold concentration did not interfere	[19]
^e^ MWCNTs/GCE	DPV	phosphate Buffer (pH 3.5)	0.25–100	0.027	5-fold concentration did not interfere	[20]
^f^ PGA/CNTPE	^n^ CV	phosphate buffer (pH 6.0)	0.05–100	0.01	not mentioned	[21]
^g^ MWCNTs/CPE	Amperometry	phosphate buffer (pH 3.0)	0.6–9.0; 10.0–100	0.033	seriously interfered	[22]
^h^ PSA/GCE	DPV	phosphate buffer (pH 3.5)	0.05–10	0.0068	not mentioned	[23]
^i^ EGPU-tAuNP	DPV	Britton-Robinson buffer (pH 7.4)	0.6–2.0	0.053	not mentioned	[24]
^j^ ß-CD/MWCNTs/GCE	DPV	phosphate buffer (pH 3.0)	1.5–30.5	0.07	4-fold concentration did not interfere	[25]
^k^ rGO/SnO_2_/GCE	DPV	phosphate buffer (pH 7.0)	1–100	0.04	30-fold concentration did not interfere	[26]
^l^ Ta_2_O_5_-rGO-GCE	second-order derivative LSV	phosphate buffer (pH 6.0)	1–8; 8–80; 80–800	0.87	N.A	[27]
MIP/ABPE	second-order derivative LSV	phosphate buffer (pH 3.0)	0.01–4; 4–20; 20–100	0.008	20-fold concentration did not interfere	This work

^a^ butyrylcholine modified glassy carbon electrode; ^b^ gold nanoparticles modified glassy carbon electrode; ^c^ gold nanoparticles decorated multiwalled carbon nanotube modified glassy carbon electrode; ^d^ electrochemically treated pencil graphite electrode; ^e^ acid-treated multi-walled carbon nanotubes modified glassy carbon electrode; ^f^ poly-glutamic acid film modified carbon paste electrode; ^g^ multi-walled carbon nanotube modified carbon paste electrode; ^h^ Poly(sulfosalicylic acid) modified glassy carbon electrode; ^i^Gold nanoparticles modified graphitepolyurethane composite electrode; ^j^ ß-cyclodextrin incorporated with multi-walled carbon nanotube modified glassy carbon electrode; ^k^ reduced graphene oxide decorated with tin oxide nanoparticles modified glassy carbon electrode; ^l^ Ta_2_O_5_-reduced graphene oxide electrode; ^m^ differential pulse voltammetry; ^n^ cyclic voltammetry; ^o^ linear sweep voltammetry.

**Table 2:** The results for the determination of Trp in compound amino acid injections (*n* = 4).

Sample ID	Label Values/g L^−1^	Found/gL^−1^	RSD/%	Recovery/%
A^a^	0.430	0.438	2.4	102.4
B^a^	0.700	0.695	2.8	98.6
C^a^	0.900	0.913	3.1	97.3
D^b^	1.000	0.988	2.2	101.8

^a^ The sample was obtained from Xuzhou, the Fifth Pharmaceutical Corporation. ^b^ The sample was obtained from Guangzhou Green Cross Pharmaceutical Corporation.

**Table 3:** The results for the determination of Trp in blood serum samples (*n* = 4).

Sample ID	Found/µM	RSD/%	Added/µM	Total Found/µM	Recovery/%
A	2.67	2.7	3.0	5.74	102.3
B	3.45	2.3	4.0	7.52	101.8
C	4.28	2.5	4.0	8.45	104.2
D	3.37	2.9	3.0	6.32	98.3

## Supplementary Materials

The following are available online at https://www.mdpi.com/2218-273X/9/7/294/s1, Table S1. Influence of coexisting substances on the determination of 10 μM Trp; Table S2. Reusability of MIP/ABPE; Table S3. Reproducibility of MIP/ABPE; Table S4. The storage stability of MIP/ABPE.
