# Supplementary material for: A Simple and Efficient Molecularly Imprinted Electrochemical Sensor for the Selective Determination of Tryptophan
Source: Biomolecules. 2019 Jul 22;9(7):294. doi: 10.3390/biom9070294 (PMC6680830; doi:10.3390/biom9070294)
Supplement: Supplementary file 1 [file biomolecules-09-00294-s001.pdf]

Electronic Supplementary Material

Simple and Efficient Molecularly Imprinted Electrochemical Sensor for Selective Determination of Tryptophan

Table S1. Influence of coexisting substances on the determination of 10 μM Trp

Coexisting substance	Concentration / mM	Change of peak current / %	Coexisting substance	Concentration / mM	Change of peak current / %
Na^{+}	1.0	3.27	glycine	0.01	1.24
K^{+}	1.0	-1.65	alanine	1.0	0.84
Mg^{2+}	1.0	1.23	valine	1.0	0.54
Cu^{2+}	1.0	0.86	leucine	1.0	-1.25
Ca^{2+}	1.0	0.52	isoleucine	1.0	1.12
Al^{3+}	1.0	2.87	phenylalanine	1.0	0.83
Pb^{2+}	1.0	-1.48	histidine	1.0	0.35
Cl^{-}	1.0	1.52	aspartic acid	1.0	0.97
NO_3^{-}	1.0	2.37	glutamic acid	1.0	1.25
SO_4^{2-}	1.0	-1.12	lysine	1.0	2.04
oxalic acid	0.1	3.46	arginine	1.0	1.16
citric acid	1.0	-2.75	serine	1.0	0.57
glucose	1.0	1.84	threonine	1.0	0.34
lactic acid	1.0	2.35	cysteine	1.0	0.31
tartaric acid	1.0	-1.17	proline	1.0	0.24

Table S2. Reusability of MIP/ABPE

Determination times	1	2	3	4	5	6	7
Peak current / μA	2.075	2.045	2.112	2.050	2.015	2.057	2.108
Relative standard deviation (RSD)				1.70%			

Table S3. Reproducibility of MIP/ABPE

Electrode number	1	2	3	4	5	6	7	8
Peak current / μA	2.048	2.171	2.265	2.017	2.190	2.035	2.148	2.187
Relative standard deviation (RSD)				4.18%				

Table S4. The storage stability of MIP/ABPE

Day	1	2	3	4	5	6	7
Peak current / μA	2.065	2.153	2.049	2.127	2.036	2.064	2.083
Day	8	9	10	11	12	13	14
Peak current / μA	2.051	2.024	2.017	2.029	2.018	2.014	1.987
Day	15	16	17	18	19	20	
Peak current / μA	1.957	1.923	1.915	1.908	1.894	1.875	
